# B Cell Antigen Presentation Promotes Th2 Responses and Immunopathology during Chronic Allergic Lung Disease

**DOI:** 10.1371/journal.pone.0003129

**Published:** 2008-09-03

**Authors:** Dennis M. Lindell, Aaron A. Berlin, Matthew A. Schaller, Nicholas W. Lukacs

**Affiliations:** Department of Pathology, University of Michigan, Ann Arbor, Michigan, United States of America; Yale University School of Medicine, United States of America

## Abstract

**Background:**

The role of B cells in allergic asthma remains undefined. One mechanism by which B cells clearly contribute to allergic disease is via the production of specific immunoglobulin, and especially IgE. Cognate interactions with specific T cells result in T cell help for B cells, resulting in differentiation and immunoglobulin secretion. Proximal to (and required for) T cell-dependent immunoglobulin production, however, is antigen presentation by B cells. While interaction with T cells clearly has implications for B cell function and differentiation, this study investigated the role that B cells have in shaping the T cell response during chronic allergic lung disease.

**Methodology/Principal Findings:**

In these studies, we used a clinically relevant mouse model of chronic allergic lung disease to study the role of B cells and B cell antigen presentation in this disease. In these studies we present several novel findings: 1) Lung B cells from chronically allergen challenged mice up-regulated MHC II and costimulatory molecules CD40, CD80 and CD86. 2) Using *in vitro* studies, B cells from the lungs of allergen challenged mice could present antigen to T cells, as assessed by T cell proliferation and the preferential production of Th2 cytokines. 3) Following chronic allergen challenge, the levels of Th2 cytokines IL-4 and IL-5 in the lungs and airways were significantly attenuated in B cell −/− mice, relative to controls. 4) B cell driven Th2 responses and mucus hyper secretion in the lungs were dependent upon MHC II expression by B cells.

**Conclusions/Significance:**

Collectively, these results provide evidence for antigen presentation as a novel mechanism by which B cells contribute to chronic allergic disease. These findings give new insight into the mechanisms by which B cells promote asthma and other chronic diseases.

## Introduction

In recent decades, the prevalence of asthma in the US and other industrialized countries has increased dramatically [1], [2], [3]. Although some recent data suggest that the incidence of asthma may have reached a plateau [4], [5], it remains a major public health concern. Chronic allergic lung disease (allergic asthma) accounts for a majority of asthma. The disease is variable, but is characterized by pulmonary inflammation, eosinophilia, and mucus hyper secretion, leading to impaired lung function. Therapeutic options for allergic asthma include corticosteroids, antihistamines, and eicosanoid inhibitors. Recent developments in molecular therapies, including monoclonal anti-IgE, provide substantial benefit potential, but have limitations based on the cost of therapy [6]. The responsible use of these, as well as the development of other novel therapies, depends upon a proper understanding of the mechanisms which contribute to the development of allergic disease.

Many studies have demonstrated that Th2 cytokines play an important role in the development of allergic lung disease and the downstream events, including inflammation, eosinophilia, mast cell accumulation/activation, and airway remodeling [7], [8], [9], [10], [11], [12], [13], [14]. However, less is known about the mechanisms which affect the development and maintenance of Th2 cells. The initiation of pulmonary immune responses begins with recognition of antigen by pulmonary antigen presenting cells (APC), which traffic to lung associated lymph nodes. APCs in the lymph nodes prime initial T cell responses, which proliferate and traffic to the lungs. The mechanisms responsible for the perpetuation of allergic disease are poorly understood, although these likely represent a balance between pro-inflammatory signals such as infection or allergen exposure, countered by anergic and tolerogenic mechanisms [15], [16].

The role of B cells in the development and maintenance of allergic disease remains unresolved. Previous studies concerning B cells in allergic disease have concluded that B cells play no role in the development of allergic disease as characterized by inflammation, eosinophilia, and AHR [17], [18]. Other studies have suggested that the contribution of B cells is primarily via IgE [19], [20]. Still other studies which support a role for B cells in allergic lung disease did not directly address the mechanism by which B cells contribute to allergic disease [21], [22]. Significant numbers of B cells are recruited to the lungs during chronic allergic lung disease. In these studies, we sought to determine the role of B cells in the pathogenesis of chronic allergic disease, with specific attention to the role of B cells as antigen-presenting cells.

## Results

### Accumulation of B cells at the sites of inflammation during allergen-induced chronic lung disease

**Figure 1:**
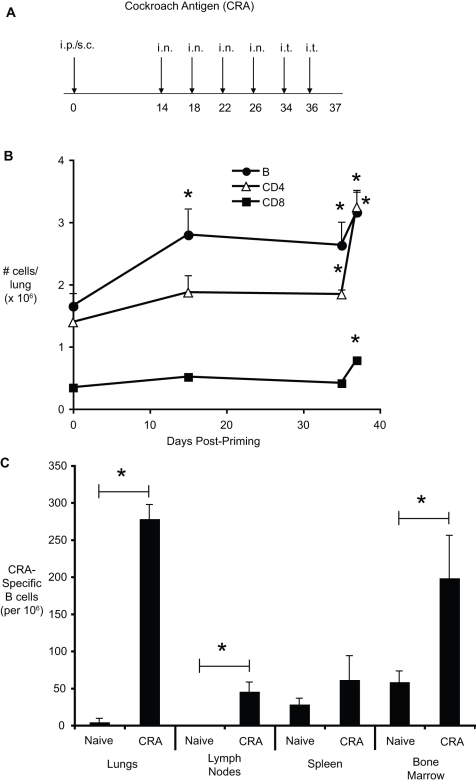
Accumulation of B cells in the lungs during chronic allergic lung disease. (A) Chronic model of cockroach antigen-induced allergic lung disease. Mice were immunized intraperitoneally (i.p.) and subcutaneously (s.c.), followed by 4 intranasal (i.n.) challenges, and two intratracheal (i.t.) inoculations. (B) Kinetics of B and T cell accumulation in the lungs of allergen challenged mice. The numbers of CD4^+^, CD8^+^, and CD19^+^ cells were determined by flow cytometric analysis of enzymatically digested lungs. Each time point represents the mean of 5 mice per group ±SEM * = p<0.05 vs. naïve lung . Similar results were observed in two independent experiments. (C) Cockroach antigen-specific B cells in the lungs, lymph nodes, spleen, and bone-marrow of naïve and allergen challenged (day 37) mice. The frequency of specific B cells was determined by a combination of ELISPOT and flow cytometry, as described in Materials and Methods. Bars represent the mean of 5 mice per group ±SEM * = p<0.05 vs. naïve. Similar results were observed in two independent experiments.

Our first objective in these studies was to determine whether B cells are a significant component of the inflammation associated with chronic allergic disease. To this end, mice were chronically challenged with cockroach antigen as detailed in Fig. 1A. At various time points following initial challenge, the numbers of CD19^+^ B cells, CD4^+^ T cells, and CD8^+^ T cells in the lungs were determined by flow cytometric analysis of enzymatic digests. In the lungs, nearly 1.6×10^6^ B cells were present in naïve lungs (Fig. 1B). The number of pulmonary B cells increased significantly following intranasal challenge, but following intratracheal challenge of a larger bolus of allergen, the number of pulmonary B cells increased dramatically, to over 3×10^6^ cells (3.16±0.35). The number of B cells in the lungs of chronically allergic mice was similar to the number of CD4^+^ T cells in these mice (3.24±0.24×10^6^). In comparison, very few CD8^+^ T cells accumulated in response to allergen challenge. To determine whether cockroach antigen - specific B cells were present in this inflammatory response, the frequency of CRA-specific B cells in various organs was assessed via a combination of ELISPOT analysis and flow cytometry. In response to allergen challenge, the frequency of CRA-specific B cells increased in the bone marrow, lung-draining lymph nodes, and in the lungs (Fig. 1C). Thus, B cells (both specific, and non-specific) are a major component of the pulmonary inflammation associated with chronic allergen-induced allergic disease.

### B cell costimulatory molecule expression in the lungs of chronic allergen challenged mice

**Figure 2:**
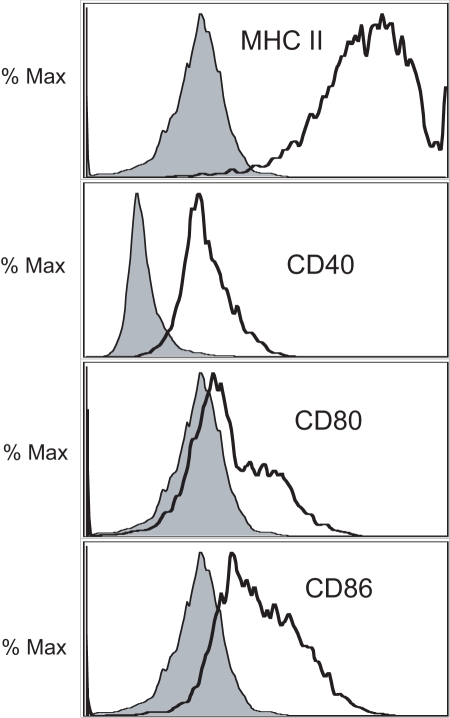
Expression of MHC II and costimulatory molecules by B cells from the lungs of chronic allergen challenged mice (day 37). Lung leukocytes were recovered from the lungs via enzymatic digests and the expression of MHC II, CD40, CD80, and CD86 on CD19^+^ cells were determined by flow cytometry. Representative histograms (from a group of n = 5) are shown. Shaded histograms represent isotype controls, with overlays representing MHC II, CD40, etc.

One mechanism by which B cells could potentially influence allergic disease is via antigen presentation. We first determined whether B cells in the lungs of allergen challenged mice express MHC II and costimulatory molecules required for stimulating infiltrating T cells. B cells were isolated from the lungs of allergen challenged mice and assayed via flow cytometry for the expression of MHC II, CD40, CD80, and CD86, as described in the Methods. B cells from the lungs of allergen challenged mice expressed high levels of MHC II, CD40, CD80, and CD86 (Fig. 2A). We next wanted to determine whether the expression of MHC II and costimulatory molecules were increased during the course of allergen challenge. To address this question, pulmonary B cell expression of MHC II and costimulatory molecules were assessed at various time points post-challenge. Following sensitization (day 13), the expression of CD40 by pulmonary B cells was increased slightly relative to B cells from naive lungs (Table 1). However, no increases in MHC II, CD80, nor CD86 were present at this time point. Similarly, at day 15 (1 day after the first intranasal challenge), the expression of MHC II and costimulatory molecules were similar to naive lung B cells. However, following chronic allergen challenge (d37), the expression of MHC II, CD80, and CD86 were dramatically up-regulated relative to B cell from the lungs of naïve mice (Table 1). Although B cells from the spleen also exhibited an increase in MHC II at day 37, the increased expression of costimulatory molecules was present only in the lungs (Table 1). These results demonstrate that B cells in the lungs of allergen-challenged mice express costimulatory molecules, as well as high levels of MHC II, consistent with a role as antigen presenting cells.

**Table 1:** B cell costimulatory molecule expression in the lungs and spleens of naive and cockroach allergen challenged mice at various time points post-challenge.

	Naive	d13	d15	d37
	**Lungs**			
**CD40**	13.33±0.36	15.30±0.12 *	14.28±0.06	15.51±0.45 **
**CD80**	9.63±0.96	8.90±0.82	8.90±0.99	15.03±2.13 *
**CD86**	21.83±0.86	24.36±1.31	20.16±1.74	55.93±5.07 **
**MHC II**	585.93±35.93	639.27±104.29	609.22±5.98	1302.33±55.07 **
	**Spleen**			
**CD40**	13.16±0.56	14.57±0.25	13.86±0.15	12.53±0.40
**CD80**	26.19±1.2	26.74±1.1	28.18±2.14	25.20±2.05
**CD86**	9.10±1.1	9.86±0.89	8.46±0.97	11.90±0.91
**MHC II**	608.10±38.6	583.07±28.13	510.51±12.56	761.70±101.2 *

Mice were euthanized at various time points during chronic allergen sensitization and challenge. Lungs and spleens were removed, enzymatically digested, and the expression of the various markers determined by flow cytometry. The median fluoresence intensity (MedFI) of CD40, etc. by CD19^+^ cells from naive, ip/sc sensitized (d13), single intranasal challenge (d15), and chronically challenged (d37) mice are shown. Bars represent the mean of 5 mice per group ±SEM. ^*^ = p<0.05 vs. naïve. ^**^ = p<0.01 vs. naïve. Similar results were observed in two independent experiments.

### Pulmonary B cells induce allergen-dependent preferential Th2 cytokine production

**Figure 3:**
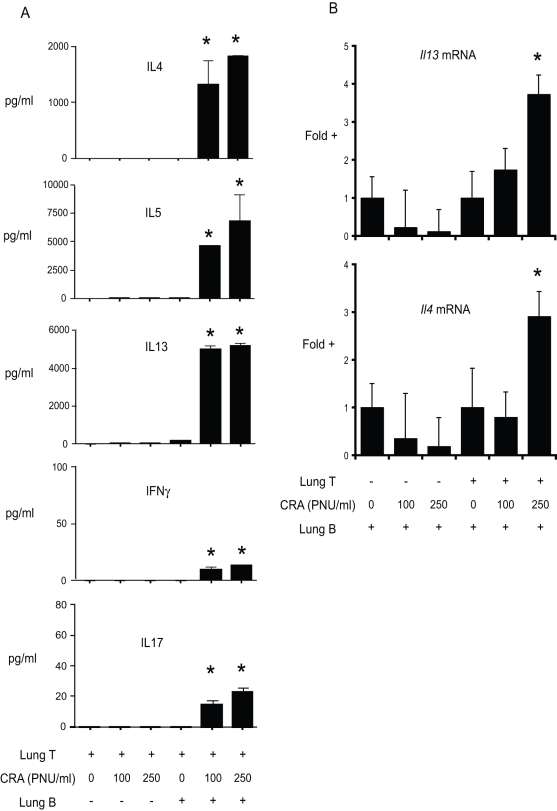
Th2 cytokine production in pulmonary B cell/T cell co-cultures. Lung lymphocytes were isolated from chronic allergen challenged mice (day 37) by enzymatic digest and enriched by density gradient centrifugation. Lung CD4^+^ T cells and CD19^+^ B cells were isolated via fluorescence activated cell sorting (FACS), and co-cultured at a ratio of 4∶1 (T∶B), with and without cockroach antigen. CD4^+^T and CD19^+^B cells were >99% pure, and contained <0.05% CD11c^+^ cells, as determined by post-sort analysis. (A) Cytokine levels in culture supernatants were determined via Bioplex-based multiplex assay after 48 hours in culture. Bars represent the mean of triplicate wells ±SEM. * = p<0.05 vs. T cell/B cell co-cultures without cockroach antigen. Similar results were observed in three independent experiments. (B) In a parallel experiment, cytokine mRNA levels were determined after 24 hour in culture via quantitative PCR. Bars represent the mean of triplicate wells ±SEM. * = p<0.05 vs. cells without antigen. Similar results were observed in two independent experiments.

Th2 cytokines play an important role in the development of chronic allergic lung disease. Our next objective was to determine whether B cells from the lungs of allergen challenged mice could present antigen to lung T cells, and determine the nature of the resulting cytokine response. In the lungs, a number of potential antigen presenting cells are present, including macrophages, dendritic cells, as well as B cells. To determine whether B cells could function directly as APCs, we purified B cells from the lungs of allergen challenged mice by FACS (>99% CD19^+^, <0.1% CD11c^+^) and determined their ability to induce antigen-dependent Th2 cytokine production by CD4^+^ T cells *in vitro*. As expected, CD4^+^ T cells alone (>99.5% pure) produced no detectable IL-4, IL-5, IL-13, IL-17 or IFNγ (Fig. 3). Upon addition of antigen, no increase in cytokine production was observed, confirming the purity of the CD4^+^ T cell isolation (Fig. 3). T cells co-cultured with B cells and antigen produced high levels of the Th2 cytokines IL-13, IL-4, and IL-5, but very little of the Th1 cytokine IFNγ or the Th17 cytokine IL-17 (Fig. 3). These data demonstrate that B cells from allergic lungs can take up, process, and present antigen to lung CD4^+^ T cells, resulting in Th2 cytokines production.

### Pulmonary B cells induce antigen-dependent T cell proliferation

**Figure 4:**
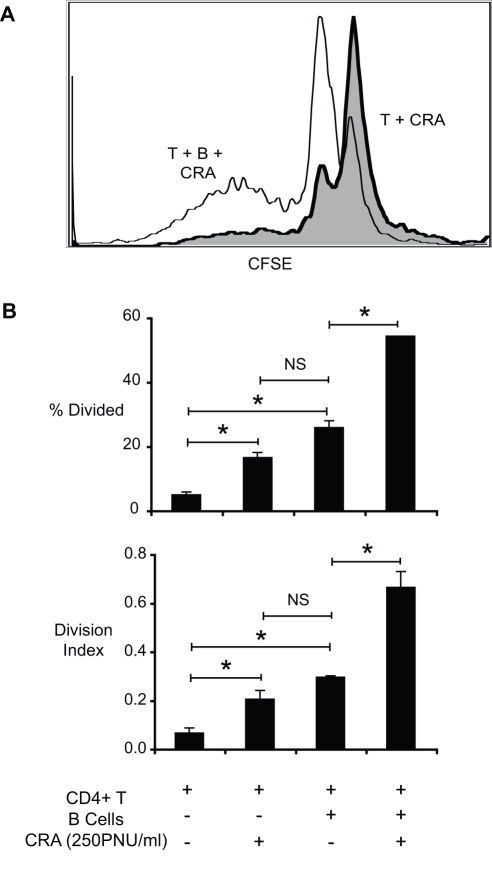
*In vitro* proliferative responses of T cells co-cultured with pulmonary B cells. Lymphocytes were isolated from chronic allergen challenged mice via enzymatic digest and density gradient centrifugation. CD4^+^ T cells and pulmonary CD19^+^ B cells were isolated via magnetic separation (MACS). Purity of isolated cell populations was >96%. CD4^+^ T cells were stained with CFSE and co-cultured with B cells with and without cockroach antigen. Cells were harvested at 72 hours post-culture, and analysed via flow cytometry. (A) Representative histogram: shaded line –T cells+CRA (250 PNU/ml), solid line – T cells+B cells+CRA (PNU/ml) (B) Percentage of dividing cells and division index were calculated using Flowjo™ software. Bars represent the mean of triplicate wells ±SEM. * = p<0.05 vs. T cells alone † = p<0.05 versus all other groups NS = not significant. Similar results were observed in two independent experiments.

To determine whether B cells from the lungs of chronically allergen challenged mice could promote T cell proliferation, CD4^+^ T cells were purified from allergen challenged mice by magnetic separation (MACS) and stained with CFSE as described in the Methods. CFSE- stained T cells were co-cultured with lung B cells with or without cockroach antigen, and the resulting proliferation was assessed via flow cytometry. Very little proliferation was observed in cultures with T cells alone (Fig. 4B), with less than 5% of the cells dividing (Fig. 4B). When cockroach antigen was added to T cells, a slight decrease in CFSE intensity was apparent (Fig. 4A), reflecting some CD4^+^ T cell proliferation (<20%, Fig. 4B). This may be due to low-level contamination of the CD4^+^ T cell preparation by APCs (purity >95% CD4^+^ T cells). Alternatively, these may represent CD4^+^ T cells dividing as a result of signals received *in vivo*. No increase in proliferation was observed upon addition of B cells without antigen (Fig 4B). In cultures with T cells, antigen, and B cells, CD4^+^ T cells exhibited a more robust proliferative response (Fig. 4A) with >50% of the cells dividing (Fig. 4B) and a proliferation index of ∼0.7 (Fig. 4B). These data demonstrate that purified lung B cells promote antigen-dependent proliferation of CD4^+^ T cells *in vitro*.

### Role of B cells in allergen-induced pathophysiology

**Figure 5:**
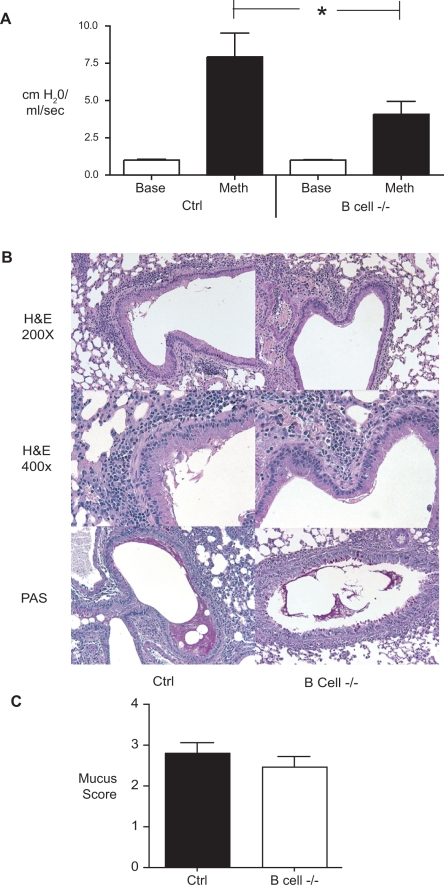
Lung pathophysiology in control and B cell −/− mice. (A) Airway hyperreactivity in control and B cell −/− mice. AHR was assessed by whole body plethysmography. The Y-axis corresponds to the increase in airway resistance observed following methacholine challenge. Bars represent the mean of 8–9 mice per group ±SEM. * = p<0.05 (B) Representative histology of lung sections from control allergen challenged and B cell−/− mice. H&E: Hemotoxylin and eosin, PAS: periodic acid Schiff's stain. (C) Histological scoring of PAS-stained lung sections. Bars represent the mean of 5 mice per group ±SEM.

Previous studies have suggested that B cell deficient mice develop inflammation and eosinophilia equivalent to wild-type mice in ovalbumin models of allergic lung disease [17], [18]. Our data suggest that B cells accumulate in the lungs dramatically over time during a more chronic allergen exposure (Fig. 1B). To determine the role of B cells in chronic allergic lung disease, we subjected B cell deficient (µMT) and wild-type mice to the protocol described in Fig. 1A. One of the hallmarks of allergic disease is airway hyperreactivity (AHR). In response to methacholine challenge, control B6 mice exhibited robust airway hyperreactivity (Fig. 5A). B cell −/− mice had significantly attenuated AHR, compared to controls (Fig. 5A). These data demonstrate that B cells contribute to allergen-induced AHR during chronic allergic lung disease. Another hallmark of allergic lung disease is the hyper secretion of mucus in the airways. To determine whether B cells contribute to inflammation and mucus hyper secretion, lung sections from B cell −/− and control mice were stained with periodic acid schiff's (PAS) or Hemotoxyolin and Eosin (H&E). Inflammation and PAS staining were equivalent between the two groups of mice (Fig. 5B, 5C). Thus, B cells contribute to airway hyperreactivity in chronic allergen-challenged mice, but the decreased AHR observed in B cell −/− mice was not associated with gross changes in inflammation or mucus hyper secretion.

### Role of B cells in influencing inflammatory cell recruitment to the lungs of allergen challenged mice

**Figure 6:**
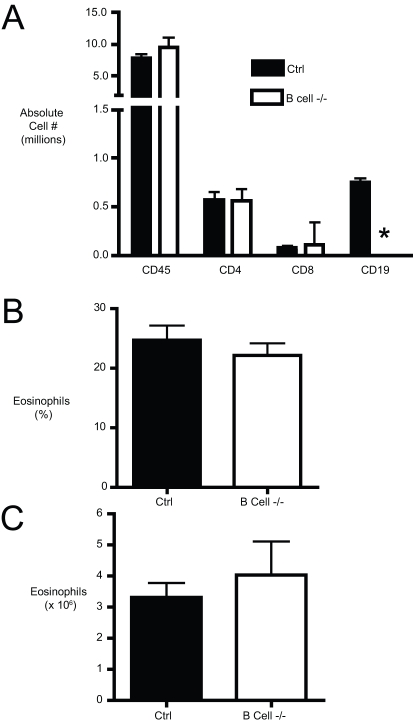
Lung leukocyte and lymphocyte recruitment to the lungs of chronic allergen challenged mice. (A) B cell −/− and control mice were chronically challenged with allergen. The numbers of CD45^+^, CD4^+^, CD8^+^, and CD19^+^ cells in the lung parenchyma were determined via flow cytometry of enzymatic digests of whole lungs. * = p<0.05 vs. control allergic mice. (B and C) Lung eosinophilia in the lungs of chronic allergen challenged mice. The frequency (B) and absolute number (C) of eosinophils in control and B cell −/− mice were determined from cytospins of enzymatically digested lungs. Bars represent the mean of 5 mice per group ±SEM. Similar results were obtained in three independent experiments.

Chronic allergic lung disease is associated with sustained inflammatory cell infiltration of the lungs. Our next objective was to determine whether B cells play a role in the magnitude or character of the inflammatory cell influx during chronic allergic disease. To this end, lungs of allergen challenged control and B cell −/− mice were enzymatically digested, and the numbers of CD45^+^, CD4^+^, CD8^+^, and CD19^+^ cells were quantified by flow cytometry, as described in the Methods. The number of CD45^+^ (or common leukocyte antigen) expressing cells in the lungs gives a quantitative measure of the magnitude of inflammation. The numbers of CD45^+^ leukocytes, as well as CD4^+^ and CD8^+^ T cells were similar between the two groups (Fig. 6A). Thus, B cells did not significantly affect the overall magnitude of the inflammatory response during chronic allergic lung disease. Chronic allergic lung disease is typically accompanied by an influx of eosinophils to the lungs. To determine whether B cells played a role in eosinophil accumulation in the lungs, the number of eosinophils in the lungs was determined via differential counts of cytospins of enzymatically digested lungs. The absolute number of eosinophils was determined by multiplying the frequency by the total cell count for that lung (obtained using a hemocytometer), as described in the Methods. No differences in eosinophilic frequency (Fig. 6B) or absolute number (Fig. 6C) were observed in the lungs of control versus B cell −/− mice. Taken together, these data demonstrate that B cells do not alter the pulmonary inflammatory cell influx during chronic allergic lung disease.

### Levels of Th1, Th2, and Th17-associated cytokines in the lungs of chronic allergen challenged mice

**Figure 7:**
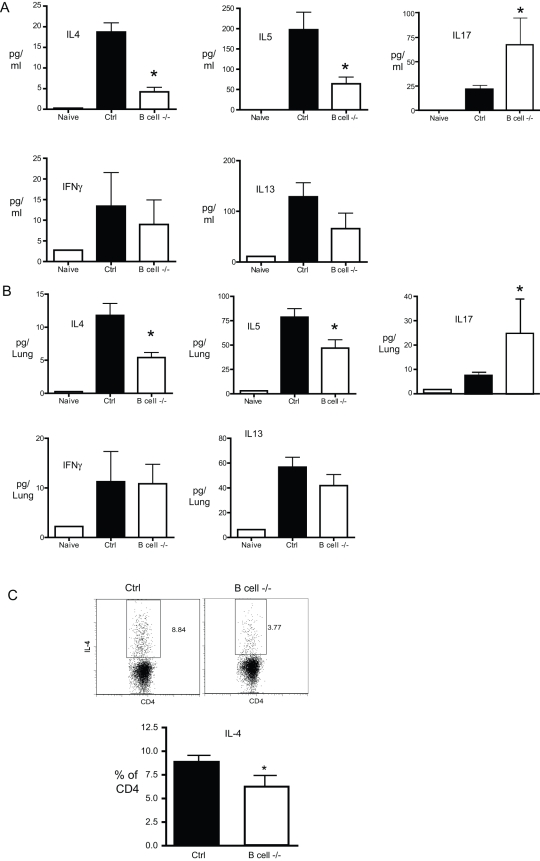
Th1/Th2 associated cytokines in the lungs of allergen challenged mice. B cell −/− and control mice were chronically challenged with allergen, and cytokine levels were assesed in bronchoalveolar lavage (B) and lung homogenates (A) via Bioplex multiplex assay. Bars represent the mean of 6 mice per group ±SEM, except naive, for which n = 2. (C) Intracellular cytokine staining of CD4 T cells from the lungs of control and B cell−/− allergen challenged mice. Bars represent the mean of 6 mice per group ±SEM. * = p<0.05 versus control allergen challenged. Similar results were obtained in two independent experiments.

The development of allergic lung disease is dependent on the production of Th2-associated cytokines IL-4, IL-5, and IL-13. The role of IL-17 in allergic disease is as yet unclear, but IL-17 may also play a role in promoting mucus hyper secretion [23], [24], [25], [26]. We next determined how B cells contribute to the production of Th1, Th2, and Th17 cytokines in the lungs of chronic allergic mice. Control and B cell −/− were chronically challenged with CRA and the levels of Th2 cytokines in the lungs were determined by analysis of whole lung homogenates and bronchoalveolar lavage by Bioplex multiplex assay. Following chronic allergen challenge, IL-4, IL-5, IL-13, and (to a lesser degree) IFNγ and IL-17 were up-regulated in the lungs and airways of control allergen challenged mice (Fig. 7A, 7B). In B cell −/− mice, the production of the Th2 cytokines IL-4 and IL-5 was significantly attenuated, in both lungs and BAL (Fig. 7A, 7B). While not absolute, the reduction in Th2 cytokines in B cell −/− mice was >60% in BAL and approximately 50% in lungs. In contrast, the levels of IL-17 was increased in B cell−/− mice, relative to controls (Fig. 7A, B). CD4^+^ T cells in the lungs of B cell −/− mice had significantly lower frequencies of IL-4 production, as assessed by intracellular cytokine staining (Fig. 7C). Interestingly, no IL-17 production by CD4^+^ T cells was detected via intracellular cytokine staining (data not shown), suggesting that IL-17 in this system is primarily non-T cell-derived. Together, these results demonstrate that B cells play a role in promoting the production of Th2 cytokines IL-4 and IL-5, but limiting IL-17 during chronic allergic lung disease.

### Role of B cell MHC II expression on the development of Th2 cytokine production and pathophysiology during chronic allergic lung disease

**Figure 8:**
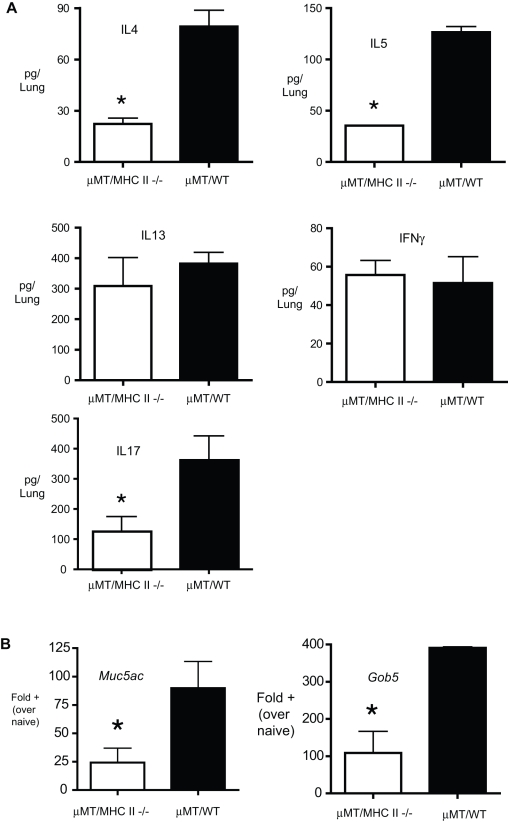
Role of B cell expression of MHC II in allergic disease. (A) Lung cytokines and (B) mucus-associated gene expression in the lungs of µMT/I-A^b^−/− mice and µMT/WT mice. Mice with MHC II deficient (µMT/I-A^b^−/− mice) or normal (µMT/WT ) B cells were generated using mixed bone marrow chimeras, and chronically challenged with allergen. The levels of cytokines in the lungs were determined from lung homogenate samples via Bioplex multiplex assay. The expression of mucus associated genes was assessed by real-time PCR using whole lung RNA. Bars represent the mean ±SEM of 4 mice per group. * = p<0.05 Similar results were obtained in two independent experiments.

To determine whether decreased Th2 cytokines and attenuated pathophysiology observed in B cell −/− mice were dependent upon antigen presentation by B cells, we used mixed bone marrow chimeras, as previously described [27]. B cell deficient µMT mice were reconstituted with a mixture of 75% µMT/25% WT bone marrow or 75% µMT/25% MHC II−/− bone marrow. Mice receiving MHC II −/− bone marrow thus receive only MHC II−/− B cells, whereas other APCs are largely normal. Mice were rested for eight weeks, then chronically challenged with allergen. The levels of Th2 cytokines in the lungs were determined by analysis of whole lung homogenates. Similar to the results obtained in Fig. 7, B cell expression of MHC II resulted in enhanced IL4 and IL-5, but not IL-13 or IFNγ (Fig. 8A). B cell expression of MHC II also increased the production of IL-17, relative to mice receiving MHC II−/− B cells. Additionally, the expression of mucus associated genes *Muc5ac* and *Gob5* were significantly increased in wild-type B cell reconstituted mice, relative to MHC II −/− controls (Fig. 8B). While not statistically significant, there was a trend towards decreased inflammatory cells in µMT/MHC II−/− chimeric mice (Table 2). These data demonstrate that B cell driven Th2 cytokine production and pathophysiology during chronic allergic lung disease is at least partially MHC-II dependent. These data strongly suggest that B cell antigen presentation contributes to the pathogenesis of chronic allergic lung disease.

**Table 2:** T cell and dendritic cell numbers in the lungs of µMT/I-A^b^−/− mice and µMT/WT mice.

Cell Type	µMT/WT	µMT/ I-A^b^−/−
CD4^+^	7.10±0.72	6.04±0.06
CD8^+^	3.32±0.41	2.53±0.35
CD11b^hi^CD11c^hi^	5.99±0.48	4.67±0.28

Mice with MHC II deficient (µMT/I-A^b^−/− mice) or normal (µMT/WT ) B cells were generated using mixed bone marrow chimeras, and chronically challenged with allergen. The percentage of each cell type was determined by flow cytometric analysis of enzymatically digested lungs, and used to calculate absolute cell numbers.

## Discussion

B cell infiltration of diseased tissues occurs in a variety of chronic diseases, including allergic lung disease, though their contribution to disease remains enigmatic. Although some other studies have demonstrated that B cells can contribute to airway hyperresponsiveness during allergic lung disease, these findings were ascribed to the production of antibodies (and IgE, in particular). Our studies are the first to provide evidence that antigen presentation by B cells contributes to the pathogenesis of allergic disease. In support of this mechanism, our studies demonstrate that: 1) Allergic disease results in the accumulation of large numbers of B cells to the lung parenchyma (a high frequency of which are antigen-specific). 2) Pulmonary B cells present antigen to T cells *in vitro*, as assessed by T cell proliferation and the production of Th2 cytokines. 3) Following chronic allergen challenge, B cell −/− mice (µMT) had attenuated airway AHR, and decreased Th2 cytokines IL-4 and IL-5. 4) Th2 cytokine production and allergic disease were attenuated in mice selectively deficient in MHC II on B cells. Thus, B cell antigen presentation plays an important role at promoting Th2 responses and pathophysiology during chronic allergic lung disease.

Although present in naïve lungs, our studies found that B cells increase dramatically in the lungs over the course of a chronic response. B cells accumulate during a number of chronic inflammatory diseases, including chronic obstructive pulmonary disease (COPD), rheumatoid arthritis (RA), and multiple sclerosis [28]. Severe COPD is often accompanied by the formation of B cell-containing lymphoid follicles surrounding airways and in the lung parenchyma, as well as B cell infiltration of airways [29], [30], [31]. Similarly, B cell follicles develop in rheumatoid synovial lesions of some patients. [32]. Normally present at very low levels in the CNS, the ratio of B cells to T cells increases dramatically in the later stages of multiple sclerosis [28]. A common theme from these studies is that B cell infiltration is associated with the most severe forms of chronic disease. The chronicity of allergen exposure in our model may explain the apparent the discrepancy between our results, and previously published studies that suggested B cells do not contribute to allergic disease [17], [18].

B cells in inflamed tissues likely serve multiple functions during chronic disease. Our studies provide *in vitro* and *in vivo* evidence that B cell antigen presentation drives Th2 cytokine production during allergic lung disease. B cell antigen presentation is becoming increasingly recognized as an important contributor to disease pathogenesis [27], [33], [34], [35], [36], [37], [38]. Previous studies supporting a role for B cells in allergic disease have concluded that the contribution of B cells is primarily via the production of IgE [19], [20]. However, specific antibodies produced by B cells in the secondary lymphoid tissues or bone marrow could presumably be transported via the circulation to the lungs, without requiring B cells to be recruited directly. Furthermore, many aspects of allergic lung disease can be generated in mast cell deficient mice, demonstrating that IgE effector functions contribute to, but are not sufficient for, the generation of allergic disease [39]. One caveat to these studies is the potential role played by Fc receptor-mediated interactions with antibodies or antibody-antigen complexes. FcR signaling can be either activating or inhibiting, and both processes play a role in regulating immune responses at mucosal surfaces [40], [41], [42]. Our studies do not discount the importance of specific antibody in the pathophysiology of allergic lung disease, but suggest that APC function of B cells is another important mechanism by which B cells contribute to disease. B cells may also contribute to allergic disease via cytokine production. Cytokine producing B cells have been described, and reported in a number of inflammatory settings [43], [44], [45], [46]. However, we did not observe direct Th2 cytokine production in our studies. Previous reports from our laboratory, as well as others, have suggested that B cells can play a regulatory role, as well [47], [48]. Still other studies have identified that B cells can indirectly influence the T cell response by altering DC function and/or lymph node organization, further modifying the resulting phenotype of the response [49], [50], [51].

In the lungs, a number of factors may influence the relative importance of B cells versus other APC in the development of T cell responses: anatomical location, intrinsic antigen uptake and processing ability, receptor specificity, and the availability of antigen. Pulmonary B cells are located predominantly in the lung parenchyma, though inflammation can be associated with infiltration of the airways. Therefore, other APCs, including dendritic cells (in the subepithelial space) and alveolar macrophages are more likely to initially encounter and capture antigen. Considerable evidence points to dendritic cells as the most efficient APC at stimulating naïve T cells [52], [53], [54], [55], [56]. However, other studies have demonstrated that B cells in lymph nodes can present antigen in levels that approximate dendritic cells [57]. Follow-up studies to this suggested that cooperation between dendritic cells and B cells takes place in the lymph nodes, with dendritic cells “handing off” antigen to the less mobile B cells [58]. Such a cooperative scenario may take place in the parenchyma of allergic lungs. Under antigen-limiting conditions, specific B cells are highly efficient at stimulating T cell responses [59]. On the other hand, under antigen non-limiting conditions, non-specific B cells can serve as potent APC [56]. Thus, the inherent disadvantages of B cells as APC (i.e. anatomic localization, poor antigen capture and processing) may be countered by the presence of: 1) specific B cells, which express high affinity antigen-specific receptors, and 2) relatively high concentrations of antigen. In the context of chronic allergic lung disease, a continuing supply of antigen, coupled with the dramatic influx of B cells to the lungs, may serve to favor B cells versus other antigen presenting cells.

The role of B cells in regulating IL-17 in allergic disease remains unclear. *In vitro* data indicated that B cells from allergic mice preferentially promoted T helper cell production of IL-4, IL-5, and IL-13, but very little IFNγ or IL-17 (Fig. 3). However, a complete lack of B cells (Fig. 7) resulted in enhanced IL-17 in the lungs. These results suggest that B cells may play a regulatory role in limiting T cell production of IL-17, or alternatively, IL-17 in the lungs may be produced by non-T cells. We did not detect T cell production of IL-17 using intracellular cytokine staining of lung lymphocytes from allergic mice, suggesting that lung IL-17 in this system is largely non-T cell-derived. In contrast, mice that received MHC II−/− B cells had decreased IL-17, relative to mice receiving WT B cells, suggesting that WT B cells were promoting IL-17 production. Thus apparent paradox may arise from compensatory mechanisms in knockout mice and/or changes to the lungs following irradiation. Although Th2 cytokines (IL-13, in particular) play a role in promoting mucus hyper secretion, high IL-17 production is also associated with mucus hyper section [23], [24], [25]. The increased IL-17 in B cell−/− mice may promote mucus hyper secretion in these mice, despite lower levels of Th2 cytokines (Fig. 7).

In conclusion, our studies are the first to show that B cells contribute to chronic allergic lung disease via promoting Th2 responses, and strongly suggest that B cell antigen presentation contributes to the pathogenesis of this disease.

## Materials and Methods

### Mice

Female µMT, I-A^b^ −/−, C57Bl/6, DO11.10, and Balb/C mice were obtained from the Jackson Laboratories. B cell deficient µMT mice are on the C57Bl/6 background. All other experiments were performed using mice on a Balb/c background. Mice were housed under pathogen-free conditions in enclosed filter-topped cages. Clean food and water were given *ad libitum*. The mice were handled and maintained using micro isolator techniques, with daily veterinarian monitoring. Bedding from the mice was transferred weekly to cages of uninfected sentinel mice that were subsequently bled at weekly intervals and found to be negative for antibodies to mouse hepatitis virus, Sendai virus, and *Mycoplasma pulmonis*. All studies involving mice were approved by the University Committee on Use and Care of Animals (UCUCA) at the University of Michigan.

### Cockroach antigen sensitization protocol

The cockroach antigen sensitization protocol is outlined in Figure 1A. Female mice (4- to 6-week-old) were immunized on day 0 intraperitoneally and subcutaneously with 1000 Protein Nitrogen Units (PNU) cockroach allergen extract (CRA, Hollister-Stier) emulsified in incomplete Freund's adjuvant (Sigma). This cockroach allergen is a skin test/immunotherapy grade preparation that has very little endotoxin contamination (<20 ng/ml) [60]. Beginning at day 14, mice received intranasal challenges of 150 PNU CRA every four days (days 14, 18, 22, 26, 30) followed by intratracheal challenges of 400 PNU on day 34 and 36.

### Measurement of airway hyperreactivity

Airway hyperreactivity (AHR) was assessed as previously described [60], [61], [62], [63], [64], [65]. AHR was measured using a Buxco mouse plethysmograph which is specifically designed for the low tidal volumes (Buxco). The mouse to be tested was anesthetized with sodium pentobarbital and intubated via cannulation of the trachea with an 18-gauge metal tube. The mouse was subsequently ventilated with a Harvard pump ventilator (tidal volume = 0.4 ml, frequency = 120 breaths/min, positive end-expiratory pressure 2.5–3.0 cm H_2_O) and the tail vein was cannulated with a 27 gauge needle for injection of the methacholine challenge. The plethysmograph was sealed and readings monitored by computer. As the box is a closed system, a change in lung volume will be represented by a change in box pressure (*P*_box_) that was measured by a differential transducer. Once baseline levels had stabilized and initial readings were taken, a methacholine challenge was given via the cannulated tail vein. After determining a dose–response curve (0.001–0.5 mg), an optimal dose was chosen, 0.250 mg of methacholine. This dose was used throughout the rest of the experiments in this study. After the methacholine challenge, the response was monitored and the peak airway resistance was recorded as a measure of airway hyperreactivity.

### Lung, lymph node, spleen, and bone-marrow leukocyte isolation

Lung leukocytes were isolated via collagenase dispersion. Right lungs from each mouse were excised, washed in PBS, minced and digested enzymatically for 40 minutes in 15 ml/lung of digestion buffer (RPMI, 5% FCS, antibiotics, 1 mg/ml collagenase (Roche Applied Science), and 30 µg/ml DNase (Sigma)). Following erythrocyte lysis using ammonium chloride (NH_4_Cl) buffer, cells were washed, and resuspended in media (RPMI, 5% FCS, antibiotics). Total lung leukocyte numbers were assessed in the presence of 0.04% trypan blue using a hemocytometer; viability was >90%. Eosinophil frequency was determined by Wright-Giemsa staining of samples cytospun onto slides. Lung-associated lymph nodes (LALN) and spleens were excised and cells dispersed with the plunger of a 3 ml syringe. Bone marrow cells were isolated via flushing femurs and tibias with PBS +1% FCS. Erythrocytes were lysed using NH_4_Cl buffer, and cells were resuspended in media. For preparation of lung lymphocytes for fluorescence-based sorting (FACS), lung leukocytes were enriched by centrifugation over a 40% isotonic Percoll® (Sigma) gradient for 30 min at 2000×g. The cell pellet was resuspended in media and plated in 100 mm cell culture dishes. Non-adherent cells were harvested and stained for FACS.

### B cell ELISPOT

High-protein binding filtration plates (Millipore) were coated with 400 PNU/ml CRA in PBS. Plates were blocked for 1 hour with 2% FCS in PBS. After washing, single cell suspensions from each organ were added at varying concentrations (5×10^5^–2×10^6^) in media, and incubated overnight at 37° 5% CO_2_. After washing away cells, spots corresponding to B cells secreting antibody were visualized using HRP conjugated anti-mouse Ig and AEC substrate, and counted using a stereo dissection microscope. Single cell suspensions from each organ varied in the frequency of B cells (∼50% in LALN, <10% in Lungs), so the frequency of spots per well was normalized to B cell number using flow cytometry by the following calculation:
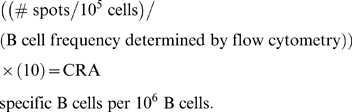



### Flow cytometry

For surface staining alone, leukocytes were washed and resuspended at a concentration of 10^7^ cells/ml in Flow Buffer ((PBS+1% FCS+0.1% NaN_3_ (Sigma)), Fc receptors were blocked by the addition of anti-CD16/32 (Fc block™, BD Biosciences). Leukocytes were stained with the following monoclonal antibodies, per manufacturer's instructions: anti-CD45 (30-F11), CD19 (1D3), CD4 (RM4-5), CD8 (53-6.7), CD40 (3/23), anti-I-A^d^ (AMS-32.1), (all from BD Biosciences), CD11c (N418), CD80 (16-10A1), CD86 (G4, all from eBiosciences). A minimum of 50,000 events were acquired on a dual-laser Cytomics FC500™ flow cytometer (Beckman Coulter) using Flowjo™ software (Treestar). The absolute number of each cell type was determined by multiplying the percentage×total cell number isolated from each organ. For fluorescence activated cell sorting (FACS), cells were stained as above, except that azide free flow buffer was used. Cells were sorted on a BD FACSAria (BD Biosciences) at the University of Michigan Flow Cytometry Core. Cells were pre-gated on CD45 to exclude epithelial cell debris, negatively gated for CD11c, and positively gated for either CD4 or CD19. Resulting cell populations were greater than 99% pure and <0.05% CD11c^+^, and greater than 95% viable as determined by trypan blue exclusion.

### Lung T cell/B cell co cultures

Lung lymphocytes were isolated from the lungs via enzymatic digestion and enriched via density gradient centrifugation as described above. The resulting cell suspension was subjected to fluorescence activated cell sorting (FACS). CD4^+^ T cells and B cells were co-cultured in complete media (RPMI, 5% FCS, antibiotics, non-essential amino acids, L-glutamine (all obtained from Mediatech, Inc.),50 mM 2-mercaptoethanol (Sigma)) at a predetermined optimal ratio of 4∶1 (200, 000 T∶50,000 B) in 96 well plates, either with or without cockroach antigen, and the levels of IL-4, IL-5, IL-13, IFNγ, and IL-17 were measured in supernatants harvested after 72 hrs.

### Intracellular flow cytometry

Leukocytes were cultured for 6 hours at 5×10^6^ cells/ml in 48-well plates in the presence of 2 µg/ml plate bound anti-CD3 (145-2C11, BD Biosciences) and Brefeldin A (in the form of Golgi-plug™, BD Biosciences). Non-adherent cells were harvested and staining for cell-surface molecules was done as described above. Cells were washed of excess surface stains, fixed and permeabilized using Cytofix/Cytoperm™ (BD Biosciences), and stained using anti-IL4 (11B11, BD Biosciences) in permeabilization buffer (FA Buffer+0.1% saponin (Sigma)) at 4°C for 30 mins. The specificity of anti-cytokine antibodies was tested by comparing staining of experimental samples to a minimum of two of three negative controls: 1) isotype control 2) excess unlabelled antibody and/or 3) pre-incubation of antibody with recombinant cytokine.

### Quantitative PCR (qPCR)

RNA was isolated from the lower left lobes of lungs by homogenization using Trizol (Invitrogen). Levels of *Gob5* and *Muc5ac* mRNA were assessed using quantitative PCR (qPCR) analysis (TaqMan) as previously described [66]. Quantification of the genes of interests were normalized to GAPDH and expressed as fold increases over naive or unstimulated controls.

### Cytokine quantification

Protein levels in the lungs were determined from homogenized upper left lobes. Protein levels of IL-4, IL-5, IL13, IL-17, and IFNγ were quantitated using commercially available X-Plex bead-based cytokine assay purchased from Bio-Rad Laboratories, and analyzed using the Bio-Plex system.

### Proliferation

*In vitro* proliferation was assayed using an *in vitro* fluorescence-based assay. MACS purified CD4^+^ T cells were stained with 5 µM 5-(and 6-) carboxyfluorescein diacetate, succinimidyl ester (CFSE, Molecular Probes) in PBS 5% FCS for 7 minutes at room temperature. Cells were washed several times to remove excess CFSE and a total of 5×10^5^ T cells were co-cultured with 1×10^5^ B cells MACS- purified B cells and CRA. A minimum of 50,000 events were acquired on a dual-laser Cytomics FC500™ flow cytometer (Beckman Coulter) and analyzed using Flowjo™ software (Treestar). The software was used to calculate percent divided, as well as division index. Division Index is the average number of cell divisions that the responding cells underwent.

### Histology

Right lobes of the lungs were isolated and immediately fixed in 10% neutral buffered formalin. Lung samples were subsequently processed, embedded in paraffin, sectioned, and placed on L-lysine-coated slides, and stained using standard histological techniques using Hemotoxylin and Eosin (H&E) and Periodic-acid Schiff (PAS). PAS staining was done to identify mucus and mucus-producing cells. Slides were scored for the intensity of PAS staining by N.W.L. blinded to the identity of the samples.

### Mixed bone-marrow chimeras

MHC II deficient B cell mice and pseudo-wild type mice were generated as previously described [27]. Briefly, B cell deficient µMT mice were lethally irradiated (1200 rads) and reconstituted with 10^7^ bone marrow cells within four hours. Mice were reconstituted with either 75% µMT bone marrow+25% wild-type bone marrow or 75% WT+25% I-Ab −/− bone marrow. Mice were rested for eight weeks before chronic allergen challenge.

### Statistical analysis

All values are means±standard error of the mean (SEM), unless otherwise indicated. Differences between two means were evaluated using the Student t test, (assuming unequal variance where dictated by F test) with p<0.05 considered to be statistically significant. Differences between greater than two groups were assessed via ANOVA with Tukey-Kramer post-test comparison.
